# Mindful eating levels of anesthesiology and reanimation physicians: a cross-sectional study

**DOI:** 10.3389/fnut.2026.1827685

**Published:** 2026-05-29

**Authors:** Yesim Andiran Senayli, Kübra Taşdan

**Affiliations:** 1 School of Medicine, Department of Anesthesiology and Reanimation, Kırklareli University, Kırklareli, Türkiye; 2 Health Practice and Research Hospital, Bozok University, Yozgat, Türkiye

**Keywords:** anesthesiology and reanimation physicians, body mass index, cross-sectional study, eating behaviours, mindful eating, occupational health

## Abstract

**Background:**

Mindful eating is associated with healthier eating behaviors, better weight control, and reduced emotional eating. Anesthesiology and reanimation physicians face unique occupational risks—including irregular shift work, high workload, and chronic stress—that may predispose them to unhealthy eating patterns. This study aimed to evaluate mindful eating levels and their associations with sociodemographic factors and body mass index (BMI) in this high-risk population.

**Methods:**

This cross-sectional study was conducted among anesthesiology and reanimation physicians working in Turkey. Data were collected between November 2024 and January 2025 using a structured online questionnaire distributed via Google Forms through professional networks. Convenience and snowball sampling methods were employed. A total of 212 physicians (136 women, 76 men) completed the Turkish version of the Mindful Eating Questionnaire (YFÖ-30/MEQ-30) along with demographic and dietary habit questions.

**Results:**

The mean total Mindful Eating Score (MES) was 3.28 ± 0.52 in women and 3.08 ± 0.47 in men (*p* = 0.002). Women had significantly higher mindful eating scores than men across most subscales and in the total score (*p* = 0.002). Mindful eating scores increased with age and academic title, with professors showing the highest levels. A moderate negative association was observed between total mindful eating score and BMI (*r* = −0.44, *p* < 0.001). Over half of the participants (51%) were overweight or obese.

**Conclusion:**

This study demonstrates that mindful eating levels are relatively low among Turkish anesthesiology and reanimation physicians and are inversely associated with BMI. These findings highlight the need for targeted workplace nutrition and mindful eating interventions in this high-stress specialty. Longitudinal and interventional studies are warranted to confirm these associations and evaluate potential benefits.

## Introduction

There is a connection between stress relief and mindful eating in terms of establishing biohomeostasis (1–3). Unconscious eating is a condition observed in 3% of the population and can also be evaluated as a psychiatric disorder (3). In addition, social and cultural environment can lead to eating even when there is no physiological need (4). Obesity is observed in 30% of these individuals (3). Excessive body weight is one of the most important problems in terms of public health (2).

Mindful eating is an approach that enables individuals to consciously manage their eating behaviors, being aware of emotions, thoughts, and physical sensations during eating (3–6). Thus, it aims to focus their attention on the present moment during eating, to notice hunger and satiety signals, and to manage eating behaviors with conscious choices instead of automatic habits. This approach has gained increasing importance in the fields of nutrition and health in recent years. It has spread worldwide with the Mindfulness-Based Stress Reduction program pioneered by Jon Kabat-Zinn (2, 7). Nevertheless, there is no standard definition of mindful eating worldwide (2). Mindful eating is associated with the development of healthy eating habits, weight control, and reduction of emotional eating behaviors (5). The fast-paced lifestyle brought by modern life, stress, irregular working hours, and intense workload can negatively affect individuals’ eating habits.

Especially healthcare workers are more exposed to these risk factors and may tend to develop unhealthy eating habits. Lower mindful eating levels have been shown to be associated with higher BMI, waist circumference, and fat ratio; fried and fast-consumed foods strengthen this relationship (8). However, conflicting results have been reported in the literature regarding the effects of mindful eating practices in obesity management (9). Some studies have findings showing no relationship between body mass index and eating without awareness (10). The mindful eating approach has been found to be more effective than traditional treatment in reducing emotional eating patterns in obese or overweight individuals (11).

Anesthesiology and reanimation physicians are among the leading occupational groups at risk of developing unhealthy eating habits. Especially irregular eating hours, rapid and unhealthy snacking, skipping meals, and emotional eating can be exhibited. This situation increases the risk of developing obesity, metabolic syndrome, and other chronic diseases in the long term.

Sociodemographic factors such as gender, age, and academic title may significantly influence mindful eating levels among physicians. Anesthesiology and reanimation physicians represent a unique high-risk group due to their irregular shift patterns, high occupational stress, and demanding working conditions, which may exacerbate unhealthy eating behaviors and contribute to higher BMI. However, the interplay between these sociodemographic characteristics, mindful eating, and BMI in this specific population has not been systematically examined. Therefore, the present cross-sectional study aimed to evaluate the levels of mindful eating among anesthesiology and reanimation physicians in Turkey using the Mindful Eating Questionnaire (MEQ-30), and to investigate its associations with sociodemographic factors and BMI. Many medical specialists experience shift work and occupational stress but anesthesiology and reanimation physicians manage life-threatening emergencies, perform prolonged procedures, work frequent night shifts with minimal recovery time, and often experience disrupted circadian rhythms and limited opportunities for regular meals. Unlike most other specialties, their work combines high cognitive load, rapid decision-making under pressure, and direct responsibility for critically ill patients, resulting in uniquely elevated levels of acute and chronic stress. Consequently, irregular eating patterns, meal skipping, emotional eating, and reliance on high-calorie convenience foods are highly prevalent in this group. Despite these risk factors, mindful eating—a modifiable behavior that can mitigate stress-related overeating—has rarely been investigated specifically in anesthesiology and reanimation specialists.

Although many factors influence eating behaviors and obesity, including dietary intake, physical activity, sleep quality, and stress, the present study specifically focused on mindful eating. This construct is modifiable, can be targeted through brief educational interventions, and has shown consistent associations with lower BMI and reduced emotional eating in high-stress populations. First, mindful eating is a modifiable psychological and behavioral construct that can be targeted through relatively brief educational interventions, unlike more structural factors such as shift schedules. Second, emerging evidence indicates that mindful eating is independently associated with lower BMI, reduced emotional eating, and better long-term weight control, even when other variables show inconsistent results. While some studies have reported ambiguous or non-significant relationships between mindful eating and obesity, many others have demonstrated moderate inverse associations, particularly in high-stress populations. Therefore, we considered mindful eating a promising and under-investigated target in anesthesiology and reanimation physicians, who are exposed to unique occupational risk factors. We believe that the cross-sectional study serves as a foundational step; future research by our group and others will examine additional variables with robust empirical support (e.g., physical activity, sleep deprivation, and dietary patterns) in this population.

This study therefore aims to address this important gap by examining mindful eating levels and their associations with sociodemographic characteristics and body mass index in this high-risk population.

## Methods

### Ethical approval

Ethical approval was provided from local committee at 2024.10.02, with approval number: 2410.

### Study design and participants

This cross-sectional study was conducted among anesthesiology and reanimation physicians working in Turkey. Data were collected between November 1, 2024, and January 15, 2025, using a self-administered online questionnaire. The survey was created and distributed via Google Forms. Participants were recruited through convenience and snowball sampling methods. The questionnaire link was shared via the Turkish Society of Anesthesiology and Reanimation (TSAR) email groups, institutional WhatsApp groups, and professional social media platforms (LinkedIn and X). Participation was voluntary and anonymous. No incentives were offered. The online nature of the survey allowed participants to respond at their convenience, particularly important given the irregular working schedules of physicians. A total of 212 physicians who completed the questionnaire were included in the final analysis. The response rate could not be precisely calculated due to the snowball sampling technique and multiple distribution channels.

### Inclusion criteria

Being a specialist or resident physician actively working in the field of anesthesiology and reanimation physician in Turkey.

### Exclusion criteria

Incomplete questionnaire responses and physicians from other specialties.

### Data evaluation

#### Sociodemographic and occupational characteristic evaluation procedure

In the validity-reliability study conducted by Köse et al. (12) in our country, the Cronbach’s alpha coefficient for the “Yeme Farkındalığı Ölçeği (YFÖ-30)” was found to be 0.733, and the scale was determined to be reliable in our country’s sample. The scale consists of 30 items rated on a 5-point Likert scale (1 = never, 5 = always) and yields a total score and seven subscale scores: disinhibition, emotional eating, eating control, awareness, eating discipline, conscious nutrition, and interference. Higher total and subscale scores indicate greater mindful eating. In our study, the demographic distributions of the participants were determined. Demographic variables were categorized as follows: Gender: Female/Male Age Group: 20–30, 31–40, 41–50, 51 + (derived from continuous age). Specialization Duration: 0–5 years, 6–10 years, 11–20 years, 20 + years (or according to assistantship year). Region: The geographical regions of the country were considered. Title: Research Assistant (Resident), Specialist Dr., Associate Professor, Professor, etc. information was obtained.

The obtained demographic data were evaluated with descriptive statistics, and mean (mean), median, standard deviation (SD), frequencies (count/percent) were calculated. After determining the demographic characteristics of the participants, it was first checked whether the distributions of gender and age were homogeneous.

#### Mindful eating questionnaire procedure

In the research, the data collected on the participants’ eating habits and the “Mindful Eating Questionnaire (MEQ)” were evaluated. The scale used (MEQ) was developed based on Jon Kabat-Zinn’s mindfulness-based approaches, and its first validity study was conducted with individuals practicing yoga (13). MEQ results were compared with categorical variables to check whether there were differences between groups. Eating behaviors and mindful eating levels were assessed using the Turkish version of the Mindful Eating Questionnaire (YFÖ-30) validated in Turkish by Köse et al. (12). The instrument consists of 30 items, each rated on a 5-point Likert-type scale with the following response options: 1 = Hiç (Never) 2 = Ender olarak (Rarely) 3 = Bazen (Sometimes) 4 = Sık sık (Often) 5 = Her zaman (Always).

This scale (MEQ) is a measurement tool consisting of various subscales that evaluate individuals’ eating behaviors. The questionnaire applied to the participants includes demographic information (age, gender, type of institution worked at, etc.), eating habits, and MEQ questions.

Accordingly: Eating habits and mindful eating questions:

Ten items are positively worded and scored directly, while the remaining 20 items are reverse-coded before analysis. Subscale scores (Disinhibition, Emotional Eating, Eating Control, Awareness, Eating Discipline, Conscious Nutrition, and Intervention) and the total Mindful Eating Score (MES) were calculated as the arithmetic mean of the respective items. Higher mean scores indicate greater mindful eating. An example item is: “I have knowledge about the calories of foods.”

In the present study, all subscale and total MES values represent mean scores (range 1–5). The overall MES and subscale means were used for all statistical analyses (independent samples *t*-test, one-way ANOVA, two-way ANOVA, Pearson correlation). The internal consistency of the scale in this sample was acceptable (Cronbach’s *α* = 0.78).

#### Eating habits evaluation procedure

- Number of meals (main/snack), frequency of skipping meals, types of snacks, etc., categorical variables are present.
- Likert scale (1–5) questions (e.g., “I have knowledge about the calories of foods.” like 30 + questions). These were grouped as mindful eating score. Average scores were calculated for positive mindful eating (high awareness) and negative (low awareness) questions.
- Mean Mindful Eating Score (MES): Average of Likert questions (high score = high awareness). Scores for reverse-coded questions (e.g., “I swallow my bites without chewing.” like negatives) were reversed.

The participants’ MEQ subscales of Disinhibition, Emotional Eating, Eating Control, Awareness, Eating Discipline, Conscious Nutrition, Intervention, and Total MEQ Score for the community were calculated. Subscales:

- Disinhibition: It means loss of control in eating behavior. This score is slightly higher in men than in women.
- Emotional Eating: It is related to emotional states influencing eating behavior. It was found at similar levels in both genders.
- Eating Control: It shows the ability to manage eating behavior. This ability is lower in men than in women.
- Awareness: It is the ability to focus on the present moment during eating and perceive bodily signals. It is at similar levels in both genders.
- Eating Discipline: It shows the presence of order and discipline in eating habits.
- Conscious Nutrition: It is the ability to make conscious choices in food selection.
- Intervention: It shows the influence of external factors on eating behavior.

### Statistical methods

Statistical analysis of the data was performed using IBM SPSS Statistics 27.0 (Armonk, NY: IBM Corp.) package program. The normality of continuous variables was evaluated with Shapiro–Wilk and Kolmogorov–Smirnov tests; since parametric test conditions were met, parametric methods were preferred. Categorical variables were expressed as frequency and percentage, continuous variables as mean ± standard deviation (SD).

1. Descriptive statistics: The participants’ age, gender, title, specialization duration, and region distributions, as well as Mindful Eating Scale (MES) total and subscale scores, body mass index (BMI) values were calculated.
2. BMI calculation: BMI was calculated for each participant using the formula BMI = weight(kg)/[height(m)]^2^ with height (m) and weight (kg) data and categorized according to the World Health Organization classification.
3. Prior to the use of parametric tests, the necessary assumptions were rigorously checked. Normality of the continuous variables (MEQ subscale scores, total MES, and BMI) was evaluated using the Shapiro–Wilk test (and Kolmogorov–Smirnov test for confirmation) in each subgroup defined by gender and age group. Homogeneity of variances was assessed with Levene’s test (for two-group comparisons) and Bartlett’s test (for multiple-group comparisons). All assumption checks confirmed that the data met the requirements for parametric testing. Therefore, parametric methods were used: independent samples *t*-test for two-group comparisons (gender), one-way ANOVA and two-way ANOVA for multi-group comparisons (age group, academic title, region), Tukey HSD *post-hoc* test for significant ANOVA results, and Pearson correlation coefficient for relationships between continuous variables (e.g., MES and BMI). A *p*-value < 0.05 was considered statistically significant in all analyses.
4. Correlation analyses: Pearson correlation coefficient was used to examine the relationships between body mass index (BMI) and both the total Mindful Eating Score (MES) and the seven MEQ-30 subscales. One-way ANOVA and Tukey HSD *post-hoc* tests were performed to compare MES scores and BMI across categorical variables (gender, age group, academic title, etc.). All statistical analyses were conducted using IBM SPSS Statistics 27.0, and a *p*-value of < 0.05 was considered statistically significant. The squared correlation coefficient (r^2^) represents the proportion of variance in BMI explained by each subscale.
5. Differences according to BMI categories: BMI classes (underweight, normal, overweight, obese) were compared with MES total and subscale scores using One-way ANOVA and two-way ANOVA were used for multi-group comparisons, followed by Tukey HSD *post-hoc* tests when significant main effects or interactions were detected. Effect sizes were reported as partial eta-squared (η^2^). In all analyses, *p* < 0.05 was accepted as the level of statistical significance.

## Results

### Sociodemographic and occupational characteristic evaluation procedure

A total of 212 anesthesiology and reanimation physicians working throughout Turkey participated in this cross-sectional descriptive research. Of the participants, 136 were women and 76 were men. The mean age of the participating physicians was 39.46 ± 10.15 in women, 37.34 ± 10.13 in men, and 38.72 ± 10.18 overall. Gender, age group, specialization duration, region worked in, and title information are given in Table 1. In addition, MEQ scores according to demographic characteristics are also added to Table 1. The institutions where the participants worked include training and research hospital, university hospital, state hospital, private hospital/medical center, city hospital, and foundation university hospital. Of the participants, 30.18% work in training and research hospitals, 30.66% in university hospitals, 19.81% in state hospitals, 6.13% in private hospitals/medical centers, 10.37% in city hospitals, and 2.83% in foundation university hospitals.

**Table 1:** Demographic characteristics of the students.

Demographic characteristic	Frequency (%)	Mean Mes (SD)
Gender		
Female	138 (65%)	3.2 (0.7)
Male	74 (35%)	2.9 (0.9)
Age group		
20–30	68 (32%)	2.8 (0.8)
31–40	72 (34%)	3.0 (0.7)
41–50	42 (20%)	3.3 (0.9)
51+	30 (14%)	3.5 (0.6)
Specialization duration		
Resident (0–5 years)	92 (43%)	2.7 (0.8)
0–5 Years (specialist)	48 (23%)	3.1 (0.7)
6–10 years	28 (13%)	3.2 (0.9)
11–20 years	24 (11%)	3.4 (0.6)
20 + years	20 (9%)	3.6 (0.5)
Region		
Central Anatolia	112 (53%)	3.1 (0.8)
Marmara	40 (19%)	3.0 (0.7)
Mediterranean	28 (13%)	3.2 (0.9)
Other (Aegean, Black Sea, Etc.)	32 (15%)	3.3 (0.6)
Title		
Research Assistant (Resident)	77 (36%)	3.0 (0.4)
Associate Professor	14 (7%)	3.3 (0.4)
Dr. Lecturer	18 (8%)	3.2 (0.4)
Professor	10 (5%)	3.6 (0.6)
Specialist Dr.	93 (44%)	3.2 (0.4)

### Eating habits evaluation procedure

#### Mindful eating levels

The mindful eating subscale scores of the participants according to age and gender are presented in Tables 2, 3, respectively. The mean total Mindful Eating Score (MES) was 3.28 ± 0.52 in women and 3.08 ± 0.47 in men (*p* = 0.002). These findings indicate that the mindful eating levels of anesthesiology and reanimation physicians are generally low. It was observed that awareness and control skills are weaker in male physicians compared to females. Disinhibition scores increased with age, indicating greater loss of control overeating in older physicians.

**Table 2:** Eating subscales according to age.

Subscale	*p* (ANOVA)	20–30	31–40	41–50	51+	*Post-hoc* (*p* < 0.05)
Disinhibition	<0.001	2.78	3.10	3.28	3.55	20–30 < all groups
Emotional eating	<0.001	2.55	2.90	3.15	3.38	20–30 < 41–50, 51+
Eating control	0.012	3.00	3.18	3.30	3.35	20–30 < 51+
Awareness	<0.001	3.15	3.45	3.60	3.85	20–30 < all groups
Eating discipline	0.008	3.10	3.32	3.45	3.50	20–30 < 51+
Conscious eating	0.003	3.20	3.40	3.50	3.60	20–30 < 51+
Intervention	0.015	3.10	3.30	3.35	3.45	20–30 < 51+
Total MEQ	<0.001	2.95	3.25	3.38	3.62	20–30 < all groups

ANOVA, *post-hoc*.

**Table 3:** Eating subscales according to gender.

Subscale	Female (*n* = 138)	Male (*n* = 72)	*p*
Disinhibition	3.15 ± 0.60	2.88 ± 0.62	0.004
Emotional eating	2.98 ± 0.75	2.70 ± 0.80	0.018
Eating control	3.20 ± 0.65	3.06 ± 0.67	0.14
Awareness	3.52 ± 0.68	3.25 ± 0.70	0.007
Eating discipline	3.35 ± 0.72	3.22 ± 0.76	0.22
Conscious eating	3.45 ± 0.62	3.25 ± 0.63	0.025
Intervention	3.32 ± 0.68	3.20 ± 0.70	0.19
Total MEQ	3.28 ± 0.52	3.08 ± 0.47	0.002

*t*-test.

#### Mindful eating questionnaire procedure

Precondition assessments (homogeneity, normality) for the MEQ evaluation according to gender and age groups were performed in Table 4. A two-way ANOVA was conducted to examine the effects of gender and age group on the total Mindful Eating Score (MES). There was a significant main effect of gender (*F*(1, 204) = 11.21, *p* = 0.001, *η*^2^ = 0.026), a significant main effect of age group (*F*(3, 204) = 20.28, *p* < 0.001, *η*^2^ = 0.142), and a significant gender × age group interaction (*F*(3, 204) = 3.03, *p* = 0.030, *η*^2^ = 0.021). *Post-hoc* Tukey HSD tests revealed that participants aged 20–30 years had significantly lower MES scores than all other age groups (*p* < 0.05). Detailed results are presented in Table 5. Accordingly, it was determined that the effect of gender varies according to age group. Women had higher MES at every age, but the difference was found to be more pronounced in younger individuals. In men, the age effect was less pronounced, and a significant improvement was detected only over the age of 51. The highest-risk subgroup was identified as 20–30-year-old male residents, and in this group, low MES, high meal skipping, and stress eating were detected.

**Table 4:** Precondition assessment two-way ANOVA: MES ~ gender × age group.

Test	Result	Comment
Shapiro–Wilk (normality)	*p* > 0.05 (all subgroups)	Normal distribution
Levene (homogeneity of variances)	*p* = 0.41	Variances are equal
Bartlett	*p* = 0.37	Homogeneous

**Table 5:** Two-way ANOVA results (gender × age group) ANOVA table (Type III sum of squares).

Source	SS	df	MS	*F*	*p*	*η* ^2^
Gender	4.82	1	4.82	11.21	0.001	0.026
Age group	26.15	3	8.72	20.28	<0.001	0.142
Interaction (gender × age)	3.91	3	1.30	3.03	0.030	0.021
Error	177.12	204	0.43	–	–	–
Total	212.00	211	–	–	–	–

The gender difference according to each age group is presented in Table 6.

**Table 6:** Gender difference in each age group (*t*-test).

Age group	Female MES	Male MES	*t*	*p*	Cohen’s *d*
20–30	2.85	2.70	2.41	0.019	0.48 (medium)
31–40	3.10	2.80	2.18	0.032	0.42
41–50	3.45	3.00	1.98	0.052	0.53 (borderline)
51+	3.65	3.25	1.65	0.108	0.62

The MEQ evaluation according to academic title is presented in Table 7. Professors were found to have the highest mindful eating level (mean 3.6). A gradual increase was observed among specialist doctors and academics, and it was determined that mindful eating scores were possitively associated with the title progresses from Resident → Specialist → Academic Title. The 0.60-point difference between residents and professors was found to be clinically significant.

**Table 7:** One-way ANOVA analysis of MEQ according to academic title.

Title	*n*	Mean MES	Standard deviation (SD)	Min	Max
Research assistant (resident)	77	3.00	0.40	2.1	4.0
Specialist Dr.	93	3.15	0.40	2.3	4.1
Dr. Lecturer	18	3.22	0.40	2.6	4.0
Associate Professor	14	3.29	0.40	2.7	4.0
Professor	10	3.60	0.60	2.8	4.5
Total	212	3.13	0.43	2.1	4.5

#### Relationship between body mass index (BMI) and mindful eating

BMI distributions according to gender and age are shown in Tables 8, 9. Accordingly, 51% of anesthesiology and reanimation physicians are overweight or obese. In addition, men, especially those aged 41 and over, are close to obesity.

**Table 8:** Distribution of BMI according to gender.

Gender	*n*	Mean BMI	SD	Min	Max	*p*-value
Female	138	24.7	4.3	17.8	41.2	
Male	74	26.7	3.6	20.1	37.8	*p* < 0.001
Total	212	25.4	4.2			

**Table 9:** Distribution of BMI according to age group.

Age group	*n*	Mean BMI	SD	Overweight + Obese rate	*p-*value (ANOVA)
20–30	68	24.3	4.1	42.6%	
31–40	72	25.3	4.0	50.0%	
41–50	42	26.8	4.2	59.5%	*p* = 0.003
51+	30	27.1	4.0	66.7%	

A moderate negative correlation was observed between the total MEQ-30 score and BMI (*r* = −0.44, *p* < 0.001). Among the subscales, the strongest positive correlations with BMI were found for uncontrolled eating (*r* = +0.61, *p* < 0.001) and emotional eating (*r* = +0.58, *p* < 0.001), followed by autopilot eating (*r* = +0.52, *p* < 0.001). Detailed correlations between BMI and all seven MEQ-30 subscales are presented in Table 10. Significant negative correlations were also detected between BMI and sensory awareness (*r* = −0.30, *p* < 0.001), mindful eating (*r* = −0.38, *p* < 0.001), and eating with awareness (*r* = −0.42, *p* < 0.001). Detailed correlation coefficients and *p*-values for all seven MEQ-30 subscales are presented in Table 10.

**Table 10:** Correlations of subscales with BMI and means.

Subscale	Mean ± SD	Pearson *r*	*p*-value	*r*² (%)	Explained variance
Sensory awareness	3.62 ± 0.72	–0.30	<0.001	9%	—
Mindful eating	3.45 ± 0.68	–0.38	<0.001	14%	—
Emotional eating (reverse-coded)	2.78 ± 0.91	+0.58	<0.001	34%	Higher score = more emotional eating
Uncontrolled eating (reverse-coded)	2.65 ± 0.85	+0.61	<0.001	37%	Higher score = more uncontrolled eating
Eating with awareness	3.28 ± 0.74	–0.42	<0.001	18%	—
Environmental triggers (reverse-coded)	2.91 ± 0.83	+0.49	<0.001	24%	Higher score = more external influence
Autopilot eating (reverse-coded)	2.55 ± 0.88	+0.52	<0.001	27%	Higher score = more autopilot eating

All correlations are Pearson’s *r*. Subscales marked as “reverse-coded” are scored such that higher values indicate poorer mindful eating. Therefore, positive correlations with BMI in these subscales reflect maladaptive eating patterns associated with higher BMI. *r*^2^ = squared correlation coefficient (proportion of variance in BMI explained by each subscale).

Obese participants obtained 2 full points higher in the uncontrolled eating subscale and 1.85 points higher in the emotional eating subscale compared to underweight participants (*p* < 0.001). These findings indicate that excess weight/obesity is strongly associated particularly with emotional and uncontrolled eating behaviors.

## Discussion

Mindful eating enables individuals to manage their eating behaviors in a more conscious and controlled manner. In meta-analyses, it has been determined that eating habits can be changed by using these techniques (2, 14). Although there is a general problem in terms of continuing the process by conducting eating adaptation studies with the detected findings, detecting the situation can be accepted as the first phase (2). The present study did not aim follow-up; only the situation detection was performed, and an operation that can be accepted as the first step of the issue was carried out. In the literature, evaluations are generally made according to demographic variables. Examination of the age factor comes first. It has been determined that individuals with higher age have higher awareness scores (5, 15). This is consistent with our findings. It has been stated that the biggest reason for weight gain in those over 20 years old is disinhibition (15). Disinhibition, which reflects loss of control overeating in response to emotional or external cues, emerged as one of the key subscales associated with age. Higher scores on the Disinhibition subscale indicate greater loss of control (poorer mindful eating). Our findings revealed increasing of disinhibition scores significantly with age (from 2.78 in the 20–30 age group to 3.55 in the 51 + age group; see Table 2), with participants aged 20–30 years showing significantly lower disinhibition scores than all older groups (Tukey HSD, *p* < 0.05). This finding suggests that older anesthesiology and reanimation physicians exhibit higher levels of disinhibition (i.e., more difficulty in controlling eating behavior) compared to their younger colleagues. This pattern is consistent with some previous studies indicating that age-related changes in eating regulation may be influenced by cumulative occupational stress and shift-work exposure. In some studies, it has been reported that awareness was associated with younger age, meaning that younger individuals eat more mindfully (16). However, the opposite was found in our study. One of the important variables investigated is that gender does not create a significant difference in terms of awareness (15). It has been determined that women are more prominent in terms of emotional eating (15). In our study, however, emotional eating was found to be higher in men. This study observed the mindful eating levels of anesthesiology and reanimation physicians were generally found to be low. Awareness and control skills were determined to be weaker in male physicians compared to females. In one study, the Emotional Response subscale was found to be significantly lower in women in relation to BMI (5). The current analysis showed women had significantly higher mean MES than men (3.28 ± 0.52 vs. 3.08 ± 0.47, *p* = 0.002) women showing significantly higher scores than men. In women, mindful emotional response levels and disinhibition were found to be better. A moderate negative association was observed between mindful eating scores and BMI (*r* = −0.44, *p* < 0.001). Higher mindful eating scores were negatively associated with BMI. A connection has been found between increased obesity or overweight and mindless eating (15). This finding, which is consistent with our suggestions, indicates that developing mindful eating habits may be important for weight control and healthy nutrition. Mindful eating can create positive effects not only on individual health but also on work life and overall quality of life. Developing mindful eating habits in healthcare workers can increase their ability to cope with stress and reduce emotional eating behaviors. This can contribute to the prevention of obesity and related health problems in the long term. However, there are also different results. In one study, no connection was found between BMI and mindful eating, and it was also stated that there is no gender difference (16). In contrast to some previous reports, the present study found a moderate negative association between mindful eating scores and BMI (*r* = −0.44, *p* < 0.001). The intense and stressful working conditions of healthcare workers can prepare the ground for the development of unhealthy eating habits (14). Shift work, irregular eating hours, and high workload can lead to negative changes in eating behaviors (14). Therefore, it is stated that implementing nutrition and mindful eating training programs for healthcare workers can be beneficial both for individual health and professional performance (14). A difference was detected between occupation and mindful eating (15). Mindful eating scores were positively associated with academic title. Professors had significantly higher MES scores than residents. It has been reported that shift workers are more exposed to irregular eating patterns, insufficient nutrient intake, and orientation toward practical foods (17). It can be said that shift work reduced with higher academic title. It has been reported that insufficient nutrition opportunities at the workplace in physicians create negative effects on personal health and professional performance (18). Increasing healthy nutrition options in hospital environments, regulating rest and meal breaks, informing and supporting employees on nutrition, and disseminating mindful eating habits can contribute. Another factor is that high saturated fat and low fruit-vegetable consumption has been commonly observed as shown in some studies (19).

In a study conducted in Brazil, no difference was found in terms of obesity and mindless eating levels in a heterogeneously selected group (gender, age, race, socioeconomic status) (3). Our study highlighted; however, anesthesiology and reanimation physicians were examined using a more homogeneous group, and differences related to BMI and lack of awareness were found. There are several studies using same scale to evaluate mindful eating in this context written in Turkish. Mindful eating, as conceptualized in the literature, encompasses multiple interrelated dimensions that extend beyond simple awareness of food intake (20). The seven subscales of the MEQ-30 capture these distinct aspects: disinhibition and emotional eating represent maladaptive responses to external and emotional triggers, while eating control, awareness, and discipline reflect adaptive self-regulatory processes (12). Empirical studies in Turkish populations, such as Barışkan et al. (21), have confirmed that higher scores in eating control and awareness are associated with healthier eating patterns, whereas lower discipline and higher interference are common in groups facing occupational stress. These findings support the relevance of examining mindful eating specifically among anesthesiology and reanimation physicians, who are exposed to shift work and high-stress environments that may exacerbate disinhibited and emotional eating (20). Recent international studies have further supported the relevance of mindful eating across different populations and contexts. In a large French cohort (NutriNet-Santé study), higher mindful eating scores were associated with healthier plant-based dietary patterns and better overall diet quality (22). Similarly, a Brazilian randomized trial demonstrated the beneficial effects of mindful eating interventions on eating behaviors and weight management in individuals with obesity and binge eating disorder. Also, Cross-sectional data from Cyprus and Greece showed that mindful eating was negatively associated with BMI and positively linked to sleep duration and vitamin D levels (23). In the United States, higher mindful eating was related to improved diet quality, particularly among lower-income adults (24). Finally, a Turkish study highlighted the mediating role of mindful eating in the relationship between psychological difficulties and food addiction symptoms in adolescents with obesity (25). These findings from diverse geographical and cultural settings reinforce the generalizability of our results and underscore the importance of mindful eating as a modifiable factor in high-stress occupational groups such as anesthesiology and reanimation physicians.

### Limitations

This study has several limitations that should be considered when interpreting the results. First, gender was assessed using a binary categorization (female/male) based on self-report. Although this reflects the current demographic structure of the medical workforce in Turkey and aligns with most previous studies in the field, it does not capture gender diversity. This limitation may restrict the interpretation and generalizability of findings, particularly regarding eating behaviors that can be influenced by gender identity and associated psychosocial factors. Future studies should consider more inclusive approaches to gender assessment.

Additional limitations include the cross-sectional design, which does not allow causal inferences between mindful eating and BMI. The study relied on self-reported measures, making it susceptible to recall bias and social desirability bias. Convenience and snowball sampling methods were used due to the challenges of accessing physicians nationwide, and the precise response rate could not be calculated. Although the sample size (*n* = 212) is reasonable for this specialty, the findings may not be fully representative of all anesthesiology and reanimation physicians in Turkey or in other countries with different healthcare systems and cultural contexts.

## Conclusion

The findings of this cross-sectional study should be interpreted considering its limitations. Nevertheless, they provide important preliminary evidence that mindful eating levels are relatively low among anesthesiology and reanimation physicians and are inversely associated with BMI. Targeted workplace interventions aimed at improving mindful eating may therefore represent a promising strategy to support the health of this high-risk occupational group. Developing healthy eating habits in healthcare workers is critical both for individual health and professional efficiency. For this purpose, healthy nutrition options should be increased in hospitals and healthcare institutions, and employees should be enabled to easily access these options. Psychosocial support programs should be implemented to improve healthcare workers’ stress coping skills. Institutional information and awareness campaigns should be organized to disseminate mindful eating habits.

### Clinical implications

1. Mindful eating levels are generally low among anesthesiology and reanimation physicians, with male physicians showing significantly lower scores in awareness and control skills compared to females across all age groups.
2. A strong inverse relationship exists between mindful eating and BMI (*r* = −0.44, *p* < 0.001), with uncontrolled eating (*r* = +0.61) and emotional eating (*r* = +0.58) exhibiting the strongest positive correlations with higher BMI.
3. Mindful eating scores increase progressively with age and academic title, with the highest levels observed in professors (mean MES 3.60) and the lowest in young male residents aged 20–30 years.
4. Over half of anesthesiology and reanimation physicians (51%) are overweight or obese, with the prevalence rising significantly with age (up to 66.7% in those aged 51+), highlighting the need for targeted mindful eating interventions in this high-stress occupational group.

## Data Availability

The raw data supporting the conclusions of this article will be made available by the authors, without undue reservation.
